# Mucosal injuries from indwelling catheters: A scoping review

**DOI:** 10.1371/journal.pone.0317501

**Published:** 2025-01-16

**Authors:** Katarina E. Göransson, Ann-Christin von Vogelsang, Gabriella Engström

**Affiliations:** 1 Department of Caring Sciences, School of Health and Welfare, Dalarna University, Falun, Sweden; 2 Department of Medicine Solna, Karolinska Institutet, Stockholm, Sweden; 3 Emergency and Reparative Medicine Theme, Karolinska University Hospital, Stockholm, Sweden; 4 Department of Clinical Neuroscience, Karolinska Institutet, Stockholm, Sweden; 5 Neurocenter, Karolinska University Hospital, Stockholm, Sweden; 6 Department of Caring Sciences, School Health and Welfare, Dalarna University, Falun, Sweden; Memorial Sloan Kettering Cancer Center, UNITED STATES OF AMERICA

## Abstract

There is currently a lack of clarity concerning the types and frequency of mucosa injuries occurring in urine bladders among patients with indwelling urine catheters that are of modern design and material. The aim of the study was to identify and present the available information regarding mucosa injuries in urine bladders among adult patients with indwelling urine catheters. The research question was: What is known about mucosa injuries in urine bladders among patients with indwelling urine catheters? A scoping review applying the patient, exposure, and outcome framework. A preliminary search was made to identify the keywords, and the selection process followed the Preferred Reporting Items for Systematic Review and Meta-Analysis flow diagram. The final search across five databases retrieved a total of 8,883 records. Eight studies from three countries were included and the studies used two main methods for collecting data. Eleven concepts to describe the injuries were identified, with a range from one to five studies using the same concept. Mucosa injuries, of which polypoid cystitis was most frequently reported, occurred in all studies, and ranged from 41% to 100% per study. The size of injured area varied between 0.5 to 2.5 cm. The posterior wall of the bladder was the most common area where injuries were found. This scoping review sheds light on the limited understanding of mucosal injuries in urine bladders among adult patients with indwelling urinary catheters. Moving forward, concerted efforts are warranted to bridge existing knowledge gaps to enhance our understanding of mucosal injuries and improve clinical outcomes for adult patients with indwelling urinary catheters. The lack of a robust scientific base for the impact of indwelling urine catheters on the urine bladder mucosa warrants future studies.

## Introduction

Over 80 years ago, Dr. Foley innovated the indwelling urine catheter design that remains in use today [1]. The Foley catheter, characterized by its balloon-inflatable tip, has become a standard in medical settings, offering a reliable method for draining urine in patients with various urological conditions. Although the texture and durability of catheters have undergone significant changes since the 1990s, the concerns regarding catheter usage raised by Beeson over 40 years ago remain relevant [2]. Beeson [2] strongly advocated against routinely using indwelling urinary catheters in hospitalized patients given their potential to cause severe medical complications.

Today, the catheters come in different sizes, and the most commonly used catheter materials are latex and silicone. These materials serve as the core structure for catheters, and various coatings are available on the market to fulfil different purposes. These coatings may have specific functions, such as protecting the patient from direct contact with the core latex material, improving ease of handling and overall comfort, or providing a barrier against potential infections [3, 4]. An indwelling catheter, particularly with a large diameter, causes mechanical irritation to the mucosa of both the urethra and the bladder. The irritation to the mucosa not only disrupts local defense mechanisms but also creates an ideal surface for the development of bacterial biofilm [5].

As we explore the widespread use of indwelling urine catheters globally, particularly among patient groups with conditions such as benign prostatic hyperplasia and neurological illness and injuries, [6] patient reports shed light on negative effects such as interference with daily life activities and instances of leakage, blockage, and pain [7]. These issues, some possibly related to catheter-associated urinary tract infection (CAUTI) with or without objective signs of CAUTI, prompt a causal need for understanding the underlying causes. Almost all patients with an indwelling urinary catheter develop CAUTI if the catheter is used for ten days or longer [8]. While the precise mechanisms behind CAUTI are not fully understood, the information of microbial biofilms on the catheter surfaces plays a significant role [4]. While most research on injuries related to indwelling urine catheter treatment is CAUTI, it is crucial to acknowledge that patients also suffer from other types of effects from the treatment, which are rarely described. Understanding these additional negative effects is crucial to improve patient outcomes and different strategies have been suggested to address them; a valve instead of a urine drainage bag has been proposed as a potential improvements in catheter care [9]. When closed, the valve allows the bladder to fill with urine, and regular opening enables periodic emptying, mimicking intermittent catheterization, which is associated with lower risk of CAUTI [10]. This approach may also help maintain bladder tone and capacity [11] and reduce bladder mucosa irritation by lifting the catheter away from the bladder wall. Additional, periodic flushing via the valve may decrease the infection and catheter blockage [12, 13]. One possibility is that some of the signs and symptoms patients report during indwelling urine catheter treatment are related to the urine bladder and its loss of capacity when it is being constantly drained [14]. There is currently a lack of clarity concerning the types and frequency of mucosa injuries occurring in urine bladders among patients with indwelling urine catheters that are of modern design and material.

The rationale for carrying out a scoping review was to determine the value of future clinical research projects around mucosa injuries in urine bladders among patients with indwelling urine catheters. The objective was therefore to identify and present the available information regarding mucosa injuries in urine bladders among patients with indwelling urine catheters. The research question was: What is known about mucosa injuries in urine bladders among patients with indwelling urine catheters?

## Materials and methods

A 3-step scoping review was conducted using empirical studies from database searches: 1) identification of relevant studies; 2) screening of identified records and examining full-text papers for inclusion or exclusion; and 3) a detailed examination of the included papers. The review applied the framework of PEO (patient, exposure, and outcome) [15] in the following way: P (adult patients, living or dead), E (indwelling urinary catheter), and O (mucosa injuries). In accordance with national ethical law, ethical approval was not sought since this review does not constitute human subjects research.

### Eligibility criteria

For studies to be eligible for inclusion in the review, they had to focus on indwelling urinary catheters among adult patients and present results on mucosa injuries in the urine bladder. Furthermore, the studies were to be published in English in peer-reviewed journals during the time period 1946–2024. Both qualitative and quantitative studies were eligible in order to allow for a broad search field. Literature reviews and case studies were not eligible. Studies presenting results only on urethra injuries or CAUTI were excluded. Studies including both urethra and urine bladder perspective were included while only data around the urine bladder was extracted. Studies around CAUTI were excluded given the lack of studies on mucosa injuries in the urine bladder among patients without CAUTI.

### Information sources

The search strategy was developed in Medline (Ovid) in collaboration with librarians at the Karolinska Institutet University Library. For each search concept, we identified relevant Medical Subject Headings (MeSH terms) and free text terms. The search was then translated, in part using the Polyglot Search Translator [16], into the other databases.

A test/initial search was carried out with keywords in Medline, during which two of the researchers went through the 300 titles, abstracts, and keywords to determine how to revise the search strategy. The librarian conducted the final search, exporting the results to EndNote and removing duplicate papers before distributing them to the researchers. No gray literature or additional sources of information were sought. The search strategy is presented in full in S1 Table.

### Search

A systematic literature search of the databases CINAHL, Cochrane, Embase, MEDLINE, and Web of Science was performed. After the original search was performed on June 1^st^ 2023, the search was updated on August 5^th^ 2024. The search processes are reported per database in S1 Table. The librarian collaborated with a colleague, engaging in a peer review process to refine the search strategies before implementing them. The search strategy was designed for Medline, and the final search strategy was converted to suit the other databases.

### Selection of sources of evidence

**Fig 1:**
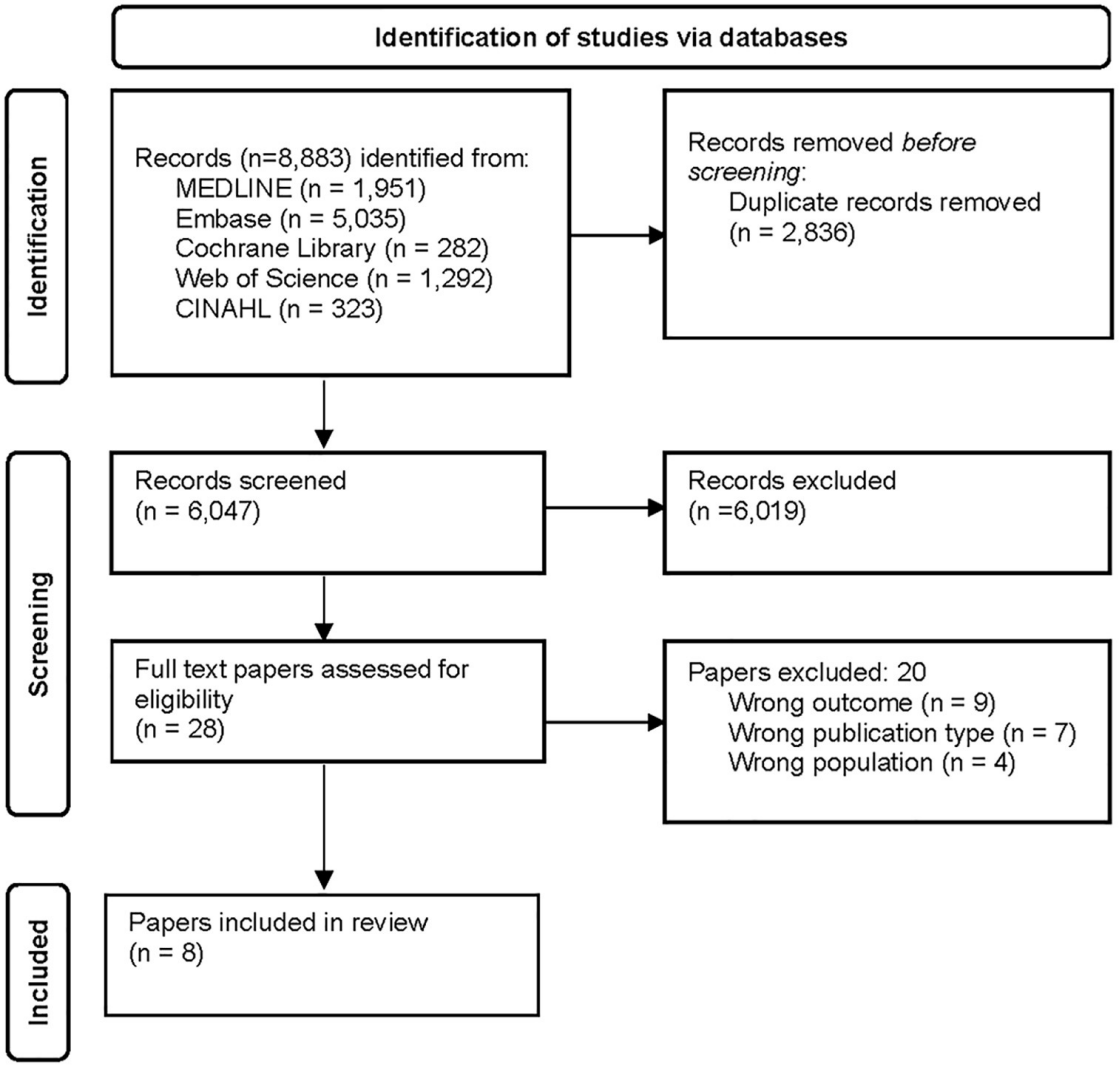
Flow diagram illustrating the identification of studies included in the review.

To ensure high quality, the selection process followed the Preferred Reporting Items for Systematic Review and Meta-Analysis (PRISMA) flow diagram [17] (Fig 1). The final search across five databases retrieved a total of 8,883 records.

After the removal of duplicate entries, we were left with 6,047 unique studies, which were exported to Rayyan. Rayyan is a specialized web-based application crafted to assist researchers when conducting reviews [18]. The subsequent step involved screening the titles and abstracts against the predefined inclusion criteria, which was performed individually by two researchers. Studies were marked as excluded (not to be included for full article assessment) or maybe (if the full text was needed to decide whether the paper was to be included or not). A total of 1,941 (32%) records were evaluated by two researchers, who were blind to each other’s assessments, and agreement was reached for 99.7% of them. After discussing the remaining four papers, consensus was reached. This step resulted in the identification of 28 papers eligible for full-text analysis. The vast majority of the records that were excluded during the screening phase focused on CAUTI.

In the next step, the 28 studies that had not been excluded were distributed across two reading pairs of researchers (one of the authors took part in both reading pairs) and were read and assessed independently. Discrepancies were solved through discussions among all authors. During the full-text analysis phase, 20 papers were excluded based on three main specific criteria (S2 Table).

### Data charting process

A data-charting form was jointly developed by all three authors to determine which variables to extract, and at what level of detail. The charting process was iterative; all authors independently charted and discussed the extracted data during this process (i.e. which headings and expressions to use when synthesizing data to enable categorisation and presentation of data in a comprehensive way). The results were discussed until consensus was reached on the final version of the data chart. Only data that were relevant to the aim were extracted.

### Data items

The data items that were extracted from the publications and were subject to critical appraisal were informed by the work of Peters and colleagues [19]. The items extracted included source of evidence (authors, title, year, and country), aim, methodology (design, data collection, grading of injuries), setting and sample, and key findings (Table 1). The key findings were further branched into specific mucosa-related findings of interest, including the type, location, and size of mucosa injuries.

**Table 1:** Characteristics of included studies.

Source of evidence	Aim	Methodology	Setting and sample	Key findings
Reference #21 Authors Abu-Yousef et al Year 1984 Country USA	To develop specific criteria that may be used to differentiate bullous (catheter) cystitis from bladder tumors	Study design Cross-sectional Method for data collection Cystosonography and cystoscopy Grading of injuries Mucosal thickening	Total number of subjects n = 23 Males/Females 23/0 Mean age 71 years (range not given) Setting Information not given Indwelling time 1 day to 6 months	Number of subjects with mucosa injuries 12/23 (52%) mucosa thickening Size of injuries Thickening from a few millimetres to 2.5 cm Areas of injuries Diffuse area: n = 6 Posterior wall: n = 6 Anterior wall: n = 2 Trigone: n = 2 Lateral wall: n = 2 Visual descriptions of the mucosa Thickened mucosa was smooth and regular in outline in the early stages. The transition from oedematous to normal mucosa was gradual. The mucosa was red or swollen and pale, no mucosal destruction associated with bullous oedema was seen. The underlying muscle wall was not involved. Additional information 8 of the 12 subjects with mucosa thickening had undergone catheter treatment ≤ 9 days while catheter treatment < 7 days did not result in mucosal thickening in 10/11 subjects.
Reference #22 Authors Ekelund & Johansson Year 1979 Country Sweden	To carry out histopathological examination of the urinary bladder	Study design Cross-sectional Method for data collection Histopathological examination of urinary bladders postmortem Grading of injuries Not specified	Total number of subjects n = 63 deceased Males/Females 34/29 Mean age 79 years (range 45–99) Setting Geriatric clinic Indwelling time < 1 month to > 24 months	Number of subjects with mucosa injuries 40/51 (78%) polypoid cystitis 23/51 (45%) von Brunn’s nest 9/51 (18%) severe inflammation with contracted bladder 7/51 (14%) diverticuli 4/51 (8%) squamous metaplasia 2/51 (4%) bladder calculi 1/51 (2%) follicular cystitis Size of injuries Macroscopical lesions (n = 12): up to 0.5 cm in diameter. Microscopical lesions (n = 28). Areas of injuries Entire bladder: n = 3 Trigone: n = 3 Posterior wall: n = 18 Dome: n = 3 Trigone + posterior wall: n = 6 Posterior wall + dome: n = 6 Trigone + posterior wall + dome: n = 1 Visual descriptions of the mucosa Urothelial hyperplasia, oedematous areas, increased ectatic vessels. Signs of recent and old haemorrhages. Additional information Organized lesions with prominent fibrosis and collagen formation were seen among subjects with urinary catheter treatment > 6 months. No normal bladders were found among subjects with catheter treatment ≥ 3 months.
Reference #26 Authors Ekelund et al Year 1983 Country Sweden	To investigate the reversibility of polypoid cystitis related to indwelling urinary catheter treatment	Study design Prospective, longitudinal Method for data collection Cystoscopy and biopsies Grading of injuries Not specified	Total number of subjects n = 20 Males/Females 11/9 Mean age 79 (range 71–89) Setting Geriatric clinic Indwelling time 1–150 months	Number of subjects with mucosa injuries 20/20 (100%) polypoid cystitis Size of injuries The lesion was in average 2 cm^2^ Areas of injuries Posterior wall or dome: n = 18 Anterior wall: n = 2 Visual descriptions of the mucosa Multiple reddened, elevated and polypoid or bullous areas of the mucosa Additional information Follow-up cystoscopies and biopsies revealed that 5 weeks post catheter removal, 8 subjects had no signs of polypoid cystitis; at 10 weeks, 2 subjects; and at 28 weeks, 3 subjects. No clear correlation between severity of the polypoid cystitis and the duration of catheter treatment was found. But the most prominent lesions were found among those with catheter treatment > 6 months. Urothelium appeared degenerative, with focal areas of necrosis located superficially, or resulting in ulcerations. The lamina propria was oedematous, with an increased number of engorged dilated vessels. Moderately severe fibrosis was seen.
Reference #23 Authors Goble et al Year 1989 Country England	A possible role for catheter-induced urothelial hyperplasia in urothelial carcinogenesis is discussed	Study design Prospective, cross-sectional Method for data collection Cystoscopy and biopsies (two biopsies were taken from each patient and were randomly labelled A or B) Grading of injuries The severity of the reaction was graded as mild, moderate, or severe (by cystoscopy) Severity of the mucosal inflammatory reaction was graded on a five-level scale (0 = no reaction to 4 = diffuse predominantly eosinophil polymorpho-nuclear infiltration in combination with intra-epithelial eosinophilic micro-abscesses) (biopsy)	Total number of subjects n = 30 Males/Females 30/0 Mean age 73 (range 58–84) Setting Information not given Indwelling time 2 days to 3 years	Number of subjects with mucosa injuries 27*/30 (90%) unspecified macroscopic injuries 3/27 (11%) absent or mild reaction 16/27 (59%) moderate reaction 11/27 (41%) severe reaction = polypoid cystitis Size of injuries Not specified Areas of injuries Most prominent on the posterior bladder wall and often involving adjacent surfaces of the collapsed bladder Visual descriptions of the mucosa A raised, oedematous, haemorrhagic papillary mucosal abnormality Additional information Histological differences between the two biopsies (A and B) were noted in 26 patients (86%) in which 8 patients without macroscopical signs of injuries had microscopical injuries found in biopsies. There was a statistically significant correlation between the grade of mucosal inflammatory response (> grade 2 vs < grade 3) and catheter indwelling time (P < 0.05). No patient with catheter indwelling time less than 1 month showed evidence of intra-epithelial micro-abscesses grade 4.
Reference #20 Authors Grocela et al Year 2010 Country USA	To investigate whether use of DOVER TM Urine Collection Systems with top vent (Tyco Healthcare Group L.P. d/b/a Couidien, Marshfield, MA, USA) top-vented urinary catheters cause fewer epithelial and vascular changes in the bladder mucosa than conventional non-vented catheters	Study design Randomized, two arms Method for data collection Cystoscopy Grading of injuries Grade 1: no lesions evident Grade 2: minor mucosal and blood vessel lesions Grade 3: major blood vessel lesions Grade 4: major mucosal and blood vessel lesions	Total number of subjects n = 41 Males/Females 0/41 Mean age 56 ± 15 years in the conventional catheter group and 60 ± 12 years in the top-vented catheter group Setting Information not given Indwelling time 16–24 hours	Number of subjects with mucosa injuries 27/41 (66%) bladder lesions Size of injuries Not specified Areas of injuries Not specified Visual descriptions of the mucosa Not specified Additional information None of the patients in the novel catheter arm exhibited mucosal changes graded as 2 or 3, whereas 14 patients in the conventional catheter arm manifested such changes. The mean change in appearance of the bladder mucosa was significantly greater in the conventional catheter arm (2.0 ± 0.23 vs. 0.4 ± 0.11, p < 0.0001).
Reference #24 Authors Norlén et al Year 1988 Country Sweden	To investigate the possible occurrence of a similar catheter induced lesion in the urethra.	Study design Cross-sectional Method for data collection Cystoscopy and biopsies Grading of injuries Not specified	Total number of subjects n = 20 Males/Females 20/0 Mean age 74 years (range 63–88 years) Setting Information not given Indwelling time 2 weeks to12 months	Number of subjects with mucosa injuries 17/20 subjects (85%) polypoid cystitis 16/16 (100%) subjects histologically verified polypoid cystitis Size of injuries Not specified Areas of injuries Posterior wall of the bladder Visual descriptions of the mucosa Reddened elevated polypoid or bullous areas Additional information Subjects without polypoid cystitis had used latex catheter (7 weeks) and silicon catheter (4 and 7 months respectively).
Reference #25 Authors Shey & Bors Year 1966 Country USA	To present the experience with pseudopapillomata of patients with spinal cord injuries and attempt to explain the etiology of this condition.	Study design Cross-sectional Method for data collection Cystoscopy and biopsies Grading of injuries Not specified	Total number of subjects n = 141 Males/Females Information not given Mean age Information not given Setting Spinal Cord Injury Center Indwelling time Information not given	Number of subjects with mucosa injuries 86/141 subjects pseudopapillomata Size of injuries Information not given Areas of injuries Information not given Visual descriptions of the mucosa Information not given Additional information Information not given
Reference #27 Authors Wall et al Year 2001 Country USA	To determine whether the inducible isoform of nitric oxide synthase is expressed in areas of chronic inflammation in the bladder of patients with spinal cord injury	Study design Longitudinal Method for data collection Cystoscopy and biopsies Grading of injuries Not specified	Total number of subjects n = 37 Males/Females 36/ 1 Mean age Men 59 years and women 62 years Setting Information not given Indwelling time > 8 years	Number of subjects with mucosa injuries 37/ 37 (100%) cystitis 20/37 (54%) squamous metaplasia 3/37 (8%) dysplastic changes 1/37 (3%) squamous cell carcinoma 36/37 (97%) positive immunostaining with anti-inducible nitric oxide synthase antibody in cells with the morphological characteristics of macrophages in areas of inflammatory infiltration in the lamina propria of bladder biopsy specimens Size of injuries Not specified Areas of injuries Not specified Visual descriptions of the mucosa N/A because no visual inspection was made Additional information Chronic inflammation varied from mild to severe, including follicular cystitis.

*excluding mild injuries

### Synthesis of results

The results from each study were individually tabled (Table 1). Thereafter, the results were narratively synthesized concerning methodology, sample composition, and mucosa injuries.

## Results

### Characteristics of the included studies

The selected studies in this scoping review were conducted in Europe (n = 3 in Sweden, n = 1 in England) and the United States (n = 4). The studies were published between 1966 and 2010, and the majority were published during the 1980s (Table 1). The included studies employed diverse study designs. One study was a randomized controlled trial, [20] and the remaining seven were of observational design: five cross-sectional studies [21–25] and two longitudinal studies [26, 27].

A total of 375 subjects were included in the eight studies, whereof one study including n = 141 subjects did not report distribution of male/female [25]. In the remaining seven studies, 234 subjects were included and 66% of the subjects were male (n = 154). Two studies exclusively included men, [21, 24] and one study included only female subjects [20]. The mean age varied across the studies, ranging from 56 years [20] to 79 years [22, 26]. Two of the studies mentioned the study setting as a geriatric clinic [22, 26] while one study was carried out at a facility for patients with spinal cord injuries [25]. For the other studies, no information on setting was given.

The duration of catheterization varied significantly across studies, ranging from 1 day to > 12 years [21, 26]. The longest catheterized period was in the prospective, longitudinal study [26]. The randomized controlled trial included subjects with an indwelling urethral or suprapubic catheter for > 8 years [27], and the two cross-sectional studies [21, 22] used inclusion catheterized periods of 1 day to 6 months and < 1 month to > 24 months respectively. The individual results of the included studies are presented in Table 1.

### Methods for observing mucosa injuries in urine bladders

The majority of the studies utilized a combination of cystoscopy and biopsies for data collection [23–27]. One study included deceased patients and collected data through histopathological examinations postmortem of entire urine bladders [22]. Other methods used were cystoscopy without additional method [20] and cystoscopy combined with cystosonography [21].

### Mucosa injuries in urine bladders among patients with indwelling urine catheters

The studies reported a variety of injury types, suggesting the presence of several distinct mucosal conditions among the subjects (Table 2). None of the studies provided very detailed information regarding the description of injury-related information, such as the manifestation of signs and the size of injuries. Even though the majority of injury types were used in several studies, as many as nine of the eleven (81%) injury types were applied by half or less of the studies.

**Table 2:** Reported injuries in the studies. # indicates reference number in the reference list.

Study	Abu-Yousef et al (1984)	Ekelund & Johansson (1979)	Ekelund et al (1983)	Goble et al (1989)	Grocela et al (2010)	Norlén et al (1988)	Shey & Bors (1966)	Wall et al (2001)
Findings	#21	#22	#26	#23	#20	#24	#25	#27
Cystoscopic, pathologic, and sonographic findings								
Bullous mucosa	x	x	x			x		
Oedematous mucosa	x	x		x				
Reddened/haemorrhagic mucosa	x	x		x		x		
Thickened mucosa	x							
Polypoid cystitis		x*	x	x		x	x**	
Mucosal lesions/ulcerations			x	x	x			
Ectatic vessels		x	x					
Histologic findings								
Lamina propria engagement		x	x					
Inflammatory response cells		x	x	x		x		x
Micro abscesses in urothelium		x	x	x		x		
Cell changes		x		x				x

*includes follicular cystitis

** includes non-neoplastic proliferations of the vesical mucosa related to polyps

### Types of mucosa injuries

The collective count of subjects diagnosed with cystitis was n = 125, thickened mucosa was observed in n = 12 cases, and bladder lesions were found in n = 27. The proportion of cystitis across the included studies varied between 41% [23] and 100% [26, 27]. This wide range underscores the significant variability in reported cystitis prevalence across studies.

Most studies provided detailed specifications regarding the types of injuries observed, e.g. reddened/haemorrhagic mucosa. However, the level of detail and specificity in reporting injury types varied considerably among studies. For example, while some studies meticulously categorized types of injuries, others provided only general or limited descriptions. Grocela [20] did not provide any information on type of injuries more specific than bladder lesions, while Abu-Yousef et al. [21] limited information to mucosa thickening. This inconsistency in reporting highlights the challenges in comparing results across studies and may obscure broader patterns or trends in injury prevalence and characteristics.

Polypoid cystitis was the predominant injury type, affecting a total of 88 subjects. This included all 40 participants in the study by Ekelund and Johansson [22], the entire cohort of 20 subjects in the study by Ekelund et al. [26], as well as 17 out of 20 subjects in the study by Norlén et al. [24], and 11 of the 30 subjects in the study by Goble et al. [23]. In the study by Shey and Bors [25] mucosa injuries were described as pseudopapillomata, defined as non-neoplastic proliferations of the vesical mucosa, closely related to infectious polyps. Mucosa thickening was reported among 12 (52%) of the 23 subjects in the study by Abu-Yousef et al. [21] and Goble et al. [23] reported haemorrhagic papillary mucosal abnormality. Inconclusive results were found regarding association between duration of indwelling urinary catheter treatment and severity of injuries, where Goble et al. [23] found such association while Ekelund et al. [26] did not.

#### Size of injuries

The reporting of injury size was not consistent across studies, with four studies omitting information on injury size [20, 23, 24, 27]. Among those studies that did provide details on injury size, measurements differed between measuring mucosa thickening [21] and the diameter or area of injured mucosa [22, 26]. The mucosa thickening varied between a few millimetres and 2.5 cm [21] and the injured area varied between 0.5 cm [22] and 2.5 cm [26].

#### Areas of injuries

Areas of injuries were thoroughly detailed in five of the studies, although the specifics varied among them [21–24, 26]. The posterior wall of the bladder, either as a singular injury or in combination with, for example, trigone and/or dome, was the most frequently reported area, consistently reported by all five studies. In addition, injuries at the anterior and lateral bladder wall were reported in three of the studies [21, 22, 26].

#### Reversibility of injuries

Ekelund et al. [26] followed up 15 subjects 28 weeks after catheter removal and found that among 13 of these subjects the polypoid cystitis had disappeared. It is noteworthy that only one study examined the reversibility of such injuries.

## Discussion

### Summary of evidence

Our literature search yielded a substantial number of records, yet studies reporting mucosal injuries in urine bladders remain relatively limited. We identified two primary methods for identifying mucosa injuries, yet there is notable variation in the concepts used to describe these injuries. The variability in reported prevalence of injuries may be related to the absence of standardized concepts. The available information appears outdated, with the most recent study published over a decade ago.

In our scoping review, we identified eight primary studies addressing mucosa injuries in urine bladders among adult patients with indwelling urine catheters. However, there is a lack of research in the field, indicating limited attention in recent years. The most recent study included in the analysis dates back to 2010, with most of the studies conducted in the 20^th^ century. The reviewed articles pose a significant analysis challenge due to changes in catheter materials [3] and to different coatings and impregnations [28, 29], which could influence the development and progression of mucosal injuries, hindering standardization across studies. This highlights a critical gap in our understanding of these potential complications associated with indwelling urinary catheter treatment.

Cystoscopy and biopsies were common data collection methods, although three studies relied exclusively on one of these methods. Standardized concepts for describing injuries are absent in the literature, in terms of findings from both cystoscopies and biopsies, which complicates comparisons across studies. The variability in reported injuries also makes it difficult to determine the true prevalence of specific injuries. For instance, it remains uncertain whether Abu-Yousef et al. [21] and Grocela et al. [20] failed to identify polypoid cystitis or if these injuries were beyond of the scope of their respective studies. Notably, none of the injuries reported in the studies were observed in all seven studies. Injury prevalence may differ due to multiple factors including inconsistency in how injuries are described and measured. However, two significant contributors to variability are indwelling time use of the catheter and the catheter material. Additionally, the material of the catheter plays a crucial role, as certain materials may be more prone to causing irritation, or bacterial colonization. Over the past decade, significant advancements have been made in catheters materials with introduction of impregnation and coatings for antimicrobial purposes, using antibiotics or antiseptic agents [30]. Besides that new materials nowadays are used (for instance polyvinyl chloride, polytetrafluoroethylene, silicone-elastomer, polymer hydromer) [31], have attempts been made to improve catheter design to prevent the catheter tip from contact with the bladder mucosa and by different balloon designs [30]. These developments make it difficult to know if the results presented in this study are transferrable to modern urinary catheters. Future research could benefit from using standardized measures for injuries to facilitate comparison across studies. One notable finding of our review is the prevalence of cystitis observed, ranging from 41% to 100%, indicating a substantial proportion of adult patients with indwelling urine catheters experience varying degrees of mucosal injuries. This emphasizes the urgent need for comprehensive preventive strategies and interventions to mitigate the risk of mucosal injuries and their associated complications.

While the exact reasons for these injuries remain unknown, prolonged catheterization duration is likely associated with an increased risk due to direct mechanical irritation from the catheter tip [32]. A notable observation from our analysis pertains to the wide range in the duration of catheterization, spanning from 1 day to over 12 years. This wide range in catheterization duration poses a challenge when attempting to draw meaningful comparisons and derive conclusive insights.

This scoping review has limitations, the main one being the few relevant studies found, despite broad and structured database searches guided by university librarians, which yielded 6,047 unique studies. However, when we conducted a test search using more restricted key words, the results were limited, leading to the result in which several of the finally included studies were not captured. The variability of study designs and measurement methods related to mucosa injuries in the included studies present another limitation.

Despite the abovementioned limitations, this review has several strengths. It is, to the best of our knowledge, the first review conducted on mucosa injuries in patients with indwelling urinary catheters, providing valuable insights into mucosa injuries in urine bladders among these patients. This review is expected to aid researchers’ understanding of the available evidence, identifying gaps in knowledge, and to serve as an initial exploration when delving into a new area of research within the field of mucosa injuries related to indwelling urine catheters.

In conclusion, our scoping review sheds light on the limited understanding of mucosal injuries in urine bladders among adult patients with indwelling urinary catheters. There is a lack of up-to-date studies and standardized concepts for describing injuries and reporting findings concerning mucosa injury in the urine bladder. To advance the field, we recommend that researchers within the field come together in joined actions aiming to develop standardized methods for identifying and defining mucosal injuries, ensuring comparability across studies.

Moving forward, concerted efforts are warranted to bridge existing knowledge gaps to enhance our understanding of mucosal injuries and improve clinical outcomes for adult patients with indwelling urinary catheters. The lack of a robust scientific base for the impact of indwelling urine catheters on the urine bladder mucosa warrants future studies.

## Supporting information

S1 Table The final search strategies reported per database. (DOCX)

S2 Table Studies excluded in the full-text analysis phase. (DOCX)

S3 Table PRISMA checklist. (DOCX)
