# Supplementary material for: Mucosal injuries from indwelling catheters: A scoping review
Source: PLoS One. 2025 Jan 16;20(1):e0317501. doi: 10.1371/journal.pone.0317501 (PMC11737791; doi:10.1371/journal.pone.0317501)
Supplement: S1 Table — (DOCX) [file pone.0317501.s001.docx]

**Supporting 1 Table.** The final search strategies reported per database.

1. Medline

| Interface: Ovid MEDLINE(R) ALL  Date of Search: 2024-08-05  Number of hits: 1951  Comment: In Ovid, two or more words are automatically searched as phrases; i.e. no quotation marks are needed  The Ovid MEDLINE®️ database contains records with the following possible status besides MEDLINE: Publisher, In-Data-Review, In-Process and PubMed-not-MEDLINE records from NLM. | Field labels   - exp/ = exploded MeSH term - / = non exploded MeSH term - .ti,ab,kf. = title, abstract and author keywords - adjx = within x words, regardless of order - * = truncation of word for alternate endings |
| --- | --- |
| Ovid MEDLINE(R) ALL <1946 to August 02, 2024>   \| 1 \| Urinary catheters/ \| 1508 \| \| --- \| --- \| --- \| \| 2 \| Catheters, Indwelling/ \| 19981 \| \| 3 \| Urinary Catheterization/ \| 14885 \| \| 4 \| ((self-retaining or Indwelling or in-dwelling or implantable or kohli or long term or Foley) adj3 catheter*).ti,ab,kf. \| 15544 \| \| 5 \| ((Bladder* or Uretera* or Urethra* or urin*) adj3 Catheter*).ti,ab,kf. \| 16512 \| \| 6 \| catheter* a demeure.ti,ab,kf. \| 8 \| \| 7 \| or/1-6 \| 52368 \| \| 8 \| exp Mucous Membrane/ \| 238636 \| \| 9 \| Cystitis/ \| 8216 \| \| 10 \| Urethritis/ \| 4769 \| \| 11 \| exp Polyps/ \| 36306 \| \| 12 \| ((Mucous* or mucos* or mucus*) adj3 (damage* or lesion* or injur* or inflammation* or wound*)).ti,ab,kf. \| 30391 \| \| 13 \| ((Bladder* or Uretera* or Urethra* or urin*) adj3 (damage* or lesion* or injur* or inflammation* or wound*)).ti,ab,kf. \| 15748 \| \| 14 \| (Cystiti* or urethriti* or polyp* or lamina propria or morphological lesion* or von brunn* nest*).ti,ab,kf. \| 386444 \| \| 15 \| or/8-14 \| 652151 \| \| 16 \| exp animals/ not humans.sh. \| 5245360 \| \| 17 \| 7 and 15 \| 2202 \| \| 18 \| 17 not 16 \| 1954 \| | |

2. Embase

| Interface: embase.com  Date of Search: 2024-08-05  Number of hits: 5035  Comment: Emtree is the controlled vocabulary in Embase | Field labels   - /exp = exploded Emtree term - /de = non exploded Emtree term - ti,ab,kw = title, abstract and author keywords - NEAR/x = within x words, regardless of order - * = truncation of word for alternate endings |
| --- | --- |
| \| No. \| Query \| Results \| \| --- \| --- \| --- \| \| #18 \| #17 NOT #16 \| 5035 \| \| #17 \| #7 AND #15 \| 5488 \| \| #16 \| [animals]/lim NOT [humans]/lim \| 6548959 \| \| #15 \| #8 OR #9 OR #10 OR #11 OR #12 OR #13 OR #14 \| 884925 \| \| #14 \| cystiti*:ti,ab,kw OR urethriti*:ti,ab,kw OR polyp*:ti,ab,kw OR 'lamina propria':ti,ab,kw OR 'morphological lesion*':ti,ab,kw OR 'von brunn* nest*':ti,ab,kw \| 474578 \| \| #13 \| ((bladder* OR uretera* OR urethra* OR urin*) NEAR/3 (damage* OR lesion* OR injur* OR inflammation* OR wound*)):ti,ab,kw \| 24733 \| \| #12 \| ((mucous* OR mucos* OR mucus*) NEAR/3 (damage* OR lesion* OR injur* OR inflammation* OR wound*)):ti,ab,kw \| 44740 \| \| #11 \| 'polyps'/exp \| 100668 \| \| #10 \| 'urethritis'/exp \| 8630 \| \| #9 \| 'cystitis'/de \| 19592 \| \| #8 \| 'mucosa'/exp \| 351891 \| \| #7 \| #1 OR #2 OR #3 OR #4 OR #5 OR #6 \| 70679 \| \| #6 \| 'catheter* a demeure':ti,ab,kw \| 11 \| \| #5 \| ((bladder* OR uretera* OR urethra* OR urin*) NEAR/3 catheter*):ti,ab,kw \| 26538 \| \| #4 \| (('self retaining' OR indwelling OR 'in dwelling' OR implantable OR kohli OR 'long term' OR foley) NEAR/3 catheter*):ti,ab,kw \| 25235 \| \| #3 \| 'bladder catheterization'/exp \| 11692 \| \| #2 \| 'indwelling catheter'/exp \| 23607 \| \| #1 \| 'urinary catheter'/exp \| 26468 \| | |

3. Cochrane Library

| Interface: Wiley  Date of Search: 2024-08-05  Number of hits: 282 | Field labels   - ti,ab,kw = title, abstract and author keywords - NEAR/x = within x words, regardless of order - * = truncation of word for alternate endings |
| --- | --- |
| \| ID \| Search \| Hits \| \| --- \| --- \| --- \| \| #1 \| [mh ^"Urinary catheters"] \| 171 \| \| #2 \| [mh ^"Catheters, Indwelling"] \| 1288 \| \| #3 \| [mh ^"Urinary Catheterization"] \| 951 \| \| #4 \| ((self-retaining:ti,ab,kw OR Indwelling:ti,ab,kw OR in-dwelling:ti,ab,kw OR implantable:ti,ab,kw OR kohli:ti,ab,kw OR "long term":ti,ab,kw OR Foley:ti,ab,kw) NEAR/3 catheter*:ti,ab,kw) \| 3995 \| \| #5 \| ((bladder*:ti,ab,kw OR urinary:ti,ab,kw OR urethra*:ti,ab,kw) NEAR/3 Catheter*:ti,ab,kw) \| 3560 \| \| #6 \| (catheter* NEXT "a demeure"):ti,ab,kw \| 0 \| \| #7 \| #1 OR #2 OR #3 OR #4 OR #5 OR #6 \| 6538 \| \| #8 \| [mh "Mucous Membrane"] \| 5765 \| \| #9 \| [mh ^Cystitis] \| 441 \| \| #10 \| [mh ^Urethritis] \| 220 \| \| #11 \| [mh Polyps] \| 1781 \| \| #12 \| ((Mucous*:ti,ab,kw OR mucos*:ti,ab,kw OR mucus*:ti,ab,kw) NEAR/3 (damage*:ti,ab,kw OR lesion*:ti,ab,kw OR injur*:ti,ab,kw OR inflammation*:ti,ab,kw OR wound*:ti,ab,kw)) \| 4882 \| \| #13 \| ((bladder*:ti,ab,kw OR urinary:ti,ab,kw OR urethra*:ti,ab,kw) NEAR/3 (damage*:ti,ab,kw OR lesion*:ti,ab,kw OR injur*:ti,ab,kw OR inflammation*:ti,ab,kw OR wound*:ti,ab,kw)) \| 1250 \| \| #14 \| (Cystiti*:ti,ab,kw OR urethriti*:ti,ab,kw OR polyp*:ti,ab,kw OR "lamina propria":ti,ab,kw OR ("morphological" NEXT lesion*):ti,ab,kw OR ("von" NEXT brunn* NEXT nest*):ti,ab,kw) \| 20392 \| \| #15 \| #8 OR #9 OR #10 OR #11 OR #12 OR #13 OR #14 \| 31061 \| \| #16 \| #7 AND #15 \| 282 \| | |

4. Web of Science Core Collection

| Interface: Clarivate Analytics  Editions = A&HCI , ESCI , SCI-EXPANDED , SSCI  Date of Search: 2024-08-05  Number of hits: 1292 | Field labels   - TS/Topic = title, abstract, author keywords and Keywords Plus - NEAR/x = within x words, regardless of order - * = truncation of word for alternate endings   Note: the *Exact search*-function was used for all the searches |
| --- | --- |
| \| # \| Search Query \| Results \| \| --- \| --- \| --- \| \| 1 \| TS=((self-retaining OR Indwelling OR in-dwelling OR implantable OR kohli OR "long term" OR Foley ) NEAR/2 catheter* ) \| 14242 \| \| 2 \| TS=((Bladder* or Uretera* or Urethra* or urin*) NEAR/2 Catheter* ) \| 14617 \| \| 3 \| TS="catheter* a demeure" \| 5 \| \| 4 \| #1 OR #2 OR #3 \| 25637 \| \| 5 \| TS=((Mucous* OR mucos* OR mucus* ) NEAR/2 (damage* OR lesion* OR injur* OR inflammation* OR wound* )) \| 30857 \| \| 6 \| TS=((Bladder* or Uretera* or Urethra* or urin*) NEAR/2 (damage* OR lesion* OR injur* OR inflammation* OR wound* )) \| 13559 \| \| 7 \| TS=(Cystiti* OR urethriti* OR polyp* OR "lamina propria" OR "morphological lesion*" OR "von brunn* nest*" ) \| 663261 \| \| 8 \| #5 OR #6 OR #7 \| 704233 \| \| 9 \| #4 AND #8 \| 1292 \| | |

5. Cinahl

| Interface: Ebsco  Date of Search: 2024-08-05  Number of hits: 323 | Field labels   - MH+ = exploded Cinahl Heading - MH = non exploded Cinahl Heading - TI = title - AB = abstract - Nx = within x words, regardless of order - * = truncation of word for alternate endings |
| --- | --- |
| \| # \| Query \| Results \| \| --- \| --- \| --- \| \| S15 \| S6 AND S14 \| 323 \| \| S14 \| S7 OR S8 OR S9 OR S10 OR S11 OR S12 OR S13 \| 51,535 \| \| S13 \| ((TI Cystiti* OR AB Cystiti*) OR (TI urethriti* OR AB urethriti*) OR (TI polyp* OR AB polyp*) OR (TI "lamina propria" OR AB "lamina propria") OR (TI "morphological lesion*" OR AB "morphological lesion*") OR (TI "von brunn* nest*" OR AB "von brunn* nest*")) \| 29,692 \| \| S12 \| ((TI Bladder* OR AB Bladder*) OR (TI Uretera* OR AB Uretera*) OR (TI Urethra* OR AB Urethra*) OR (TI urin* OR AB urin*)) N2 ((TI damage* OR AB damage*) OR (TI lesion* OR AB lesion*) OR (TI injur* OR AB injur*) OR (TI inflammation* OR AB inflammation*) OR (TI wound* OR AB wound*))) \| 2,374 \| \| S11 \| (((TI Mucous* OR AB Mucous*) OR (TI mucos* OR AB mucos*) OR (TI mucus* OR AB mucus*)) N2 ((TI damage* OR AB damage*) OR (TI lesion* OR AB lesion*) OR (TI injur* OR AB injur*) OR (TI inflammation* OR AB inflammation*) OR (TI wound* OR AB wound*))) \| 3,672 \| \| S10 \| (MH Polyps+) \| 6,286 \| \| S9 \| (MH Urethritis) \| 421 \| \| S8 \| (MH Cystitis) \| 974 \| \| S7 \| (MH "Mucous Membrane+") \| 16,346 \| \| S6 \| S1 OR S2 OR S3 OR S4 \| 9,704 \| \| S5 \| (TI "catheter* a demeure" OR AB "catheter* a demeure") \| 0 \| \| S4 \| (((TI Bladder* OR AB Bladder*) OR (TI Uretera* OR AB Uretera*) OR (TI Urethra* OR AB Urethra*) OR (TI urin* OR AB urin*)) N3 (TI Catheter* OR AB Catheter*)) \| 4,897 \| \| S3 \| (((TI self-retaining OR AB self-retaining) OR (TI Indwelling OR AB Indwelling) OR (TI in-dwelling OR AB in-dwelling) OR (TI implantable OR AB implantable) OR (TI kohli OR AB kohli) OR (TI "long term" OR AB "long term") OR (TI Foley OR AB Foley)) N3 (TI catheter* OR AB catheter*)) \| 4,115 \| \| S2 \| (MH "Urinary Catheterization") \| 2,931 \| \| S1 \| (MH "Catheters, Urinary") \| 2,309 \| | |
