# Supplementary material for: Mucosal injuries from indwelling catheters: A scoping review
Source: PLoS One. 2025 Jan 16;20(1):e0317501. doi: 10.1371/journal.pone.0317501 (PMC11737791; doi:10.1371/journal.pone.0317501)
Supplement: S2 Table — (DOCX) [file pone.0317501.s002.docx]

**Supporting 2 Table.** Studies excluded in the full-text analysis phase.

| **Reason for exclusion** | **Source of evidence (reference #)** |
| --- | --- |
| Incorrect outcome (n=9) | # 1-9 |
| Inappropriate publication type (n=7) | # 10-16 |
| Unrelated to target population (n=4) | # 17-20 |

**References**

[1] Anderson RU. Response of bladder and urethral mucosa to catheterization. Jama. 1979;242:451-453.

[2] Burton E, Gawande PV, Yakandawala N, et al. Antibiofilm activity of GlmU enzyme inhibitors against catheter-associated uropathogens. Antimicrobial agents and chemotherapy. 2006;50:1835-1840.

[3] Deng H, Wang Z, Liao L, et al. Risk Factors Predicting Upper Urinary Tract Damage in Patients With Myelodysplasia: Data Analysis of 637 Cases From A Single Center. International Neurourology Journal. 2022;26:S22-S29.

[4] Elliott TS, Reid L, Rao GG, et al. Bladder irrigation or irritation? British journal of urology. 1989;64:391-394.

[5] N A, M A S, M N, K A. Jelly belly test. Journal of the College of Physicians and Surgeons--Pakistan : JCPSP. 2006;16:434.

[6] O'Hanlan KA. Cystosufflation to prevent bladder injury. Journal of minimally invasive gynecology. 2009;16:195-197.

[7] Rao GG, Elliott TSJ. Bladder irrigation. Age and Ageing. 1988;17:373-378.

[8] Spinu A, Onose G, Daia C, et al. Intermittent catheterization in the management of post spinal cord injury (SCI) neurogenic bladder using new hydrophilic, with lubrication in close circuit devices--our own preliminary results. Journal of medicine and life. 2012;5:21-28.

[9] Stoffel JT, McGuire EJ. Outcome of urethral closure in patients with neurologic impairment and complete urethral destruction. Neurourology and urodynamics. 2006;25:19-22.

[10] Crader MF, Kharsa A, Leslie SW. Bacteriuria StatPearls. 2022.

[11] Isaacs JH, McWhorte.Dm. FOLEY CATHETER DRAINAGE SYSTEMS AND BLADDER DAMAGE. SURGERY GYNECOLOGY AND OBSTETRICS WITH INTERNATIONAL ABSTRACTS OF SURGERY. 1971;132:889-&.

[12] Sabih A, Leslie SW. Complicated Urinary Tract Infections StatPearls. 2022.

[13] Seiler WO, Stahelin HB. Practical management of catheter-associated UTIs. Geriatrics. 1988;43:43-50.

[14] Vaidyanathan S, Mansour P, Soni BM, et al. The method of bladder drainage in spinal cord injury patients may influence the histological changes in the mucosa of neuropathic bladder - a hypothesis. BMC urology. 2002;2:5.

[15] Wilde MH, Getliffe K. Urinary catheter care for older adults. Annals of Long-Term Care. 2006;14:38-42.

[16] Wu A, Aaronson D, Garcia M. Novel urethral catheter design modifications for safer outcomes. Journal of Urology. 2012;187:e799-e800.

[17] Delnay KM, Stonehill WH, Goldman H, et al. Bladder histological changes associated with chronic indwelling urinary catheter. The Journal of urology. 1999;161:1106-1109.

[18] Htoo A, George RS, Mian BM, Akgul M. Correlation of urinary catheterization with histologic grading of eosinophilic cystitis: a single institutional review of 27 cases. Academic pathology. 2023;10:100078.

[19] Lees GE, Osborne CA, Stevens JB, Ward GE. ADVERSE-EFFECTS CAUSED BY POLYPROPYLENE AND POLYVINYL FELINE URINARY CATHETERS. AMERICAN JOURNAL OF VETERINARY RESEARCH. 1980;41:1836-1840.

[20] Pfalzgraf DP, Riechardt S, Dahlem R, Fisch M. Dorsal buccal mucosa inlay for proximal penile urethroplasty - A modification of the Barbagli technique. Journal of Urology. 2009;181:13.
